# Commentary: Identification of pulmonary infections with porcine Rotavirus A in pigs with respiratory disease

**DOI:** 10.3389/fvets.2023.1102602

**Published:** 2023-01-17

**Authors:** Xia Zhou, Jia-Wei Niu, Jian-Feng Zhang, Ming Liao, Shao-Lun Zhai

**Affiliations:** ^1^Key Laboratory of Animal Disease Prevention of Guangdong Province, Department of Swine Diseases, Institute of Animal Health, Guangdong Academy of Agricultural Sciences, Ministry of Agriculture of Rural Affairs, Scientific Observation and Experiment Station of Veterinary Drugs and Diagnostic Techniques of Guangdong Province, Guangzhou, China; ^2^Maoming Branch Center of Guangdong Laboratory for Lingnan Modern Agricultural Science and Technology, Maoming, China

**Keywords:** Rotavirus A, respiratory pathogen, pulmonary infections, lung tissues, detection

## Introduction

Recently, we read significant research entitled “*Identification of pulmonary infections with porcine Rotavirus A in pigs with respiratory diseas*e” published in Frontiers in Veterinary Science, with great interest (1). We want to share our comments regarding the importance of the high detection rate of Rotavirus A in the lungs of pigs in this interesting research.

## Rotavirus: A neglected respiratory pathogen in pigs?

Generally speaking, fecal swabs and intestinal samples are candidates for detecting rotavirus, but not respiratory tissues such as the lungs, resulting in low detection rates or inaccurate test results. However, the routine was broken in a previous study (1).

## Discussion

Rotavirus (RV) is one of the most important zoonotic pathogens for humans and other animals. Pathogenically, it is one of the predominant causes of high morbidity and mortality in enteric disease in infants and young animals. RV could be divided into 10 groups (from A to J) according to the VP6 gene. Different groups could be found in different hosts. However, group A, also shown with Rotavirus A (RVA), was highly prevalent in humans, swine, and poultry (2). Epidemiologically, RV has a nearly 50-year history from the preliminary report in the State of Alaska to the present. Nowadays, RV is not only prevalent in developed countries (such as the United States, Japan, United Kingdom) but serious in a wide variety of developing countries. RV could be detected in almost species of mammals, including humans, porcine, bovine, equine, and canine. Most RV-infected cases occurred during late autumn and early winter among children between 0.5 and 3 years of age. It was reported that about 200,000 deaths are due to RV infection worldwide every year (3). Furthermore, RV can reduce the survival rates of piglets to bring huge losses to the world pig industry.

Before reading this study, we understood that RV nucleotides were frequently detected in diarrheic stool swabs. Some could be in nasopharyngeal secretions from infants with respiratory illness or neonatal piglets, rarely reported in extra-intestinal organs and tissues. However, several recent studies revealed that RVA and Rotavirus C could be detected in the lungs of diseased and healthy pigs (4, 5).

Different from other literature, the authors of this study found that the RVA-positive rates were up to 30.8% (28/91) by quantitative reverse transcription PCR. Amazingly, the number of RVA-positive intestine samples was only 8 compared to the 11 positive lung samples, indicating that the positive detection rate of the lungs is higher than the intestines in this study. The fecal swabs and intestinal samples have low detection rates or inaccurate test results of RV, the two possible reasons were as follows. On the one hand, enteric and respiratory RV infections are independent events without complete equivalent relationship between them. RV can get into the blood circulatory system causing viremia, which can reach the lungs and cause respiratory symptoms when the intestinal mucosal barrier was damaged. The previous study showed that after oral or intranasal inoculation with attenuated human RV vaccine, the number of experimental pigs with virus shedding in nasal cavity was about 10 times on average more than that of pigs with intestinal virus shedding (6). In addition, several clinical statistical studies showed that RV could be detected in nasopharyngeal secretion of infants with respiratory diseases, and further analysis showed that in children's tracheal aspirates, the detection positive rates of RV antigen (28.1%) was also higher than that with or without diarrhea symptoms accompanied by RV excretion (8.9%) (7, 8). These results indicated that the detection rates of enteric RV was lower. On the other hand, it is due to the physiological and anatomical differences between the respiratory system and enteric tract. It was reported that RV has the tendency of eosinophilic cells (6, 9). RV could be detected in nasal mucosa cells and epithelial cells of the lung, such as more and complex bronchiolar epithelial cells, goblet epithelial cells in the intestinal tract (1). In addition, there are a massive of immune cells in the lung, such as alveolar macrophage and monocyte-macrophage lineage cells. These immune cells will perform immune function and ingest some of the viral substances when substances *in vitro* invade the body, which might lead to the detection rates of respiratory RV was higher. Moreover, *in situ* hybridization, immunohistochemistry, and tissue microarray were used to prove the presence of RVA genomes and antigens in lungs, as well as transmission electron microscopy. All these two approaches showed that RVA could indeed infect the lungs. In addition, this study also showed that higher RVA infection rates occurred in pigs aged from 1 day to 8 weeks than in the lungs of pigs with unknown ages (1). A comprehensive understanding of RV infection in the lungs should be further studied.

More and more evidence shows that RVA belonging to G9 and G12 genotypes was detected in the lungs and nasal swabs associated with lung damage (10). To some extent, these studies indicated that (1) the respiratory transmission route was another route of RV transmission, (2) the lungs could be another target for RV infection, and (3) nasal swabs or lung samples should be considered candidate samples for the detection of RV.

In conclusion, the genetic diversity of RV and the increasing evidence in extra-intestinal tissues brought new challenges to scientific research and the control of RV infection. RV might be a neglected respiratory pathogen in pigs. Therefore, it is necessary to strengthen the new understanding of RV, especially including the potential mechanism of lung infection.

## Author contributions

XZ wrote the commentary. J-WN collected the data. J-FZ, ML, and S-LZ modified the manuscript. All authors read and agreed to the submission of the General Commentary.
